# Anchored PKA synchronizes adrenergic phosphoregulation of cardiac Ca_v_1.2 channels

**DOI:** 10.1016/j.jbc.2024.107656

**Published:** 2024-08-10

**Authors:** Lipeng Wang, Yi Chen, Jin Li, Ruth Westenbroek, Travis Philyaw, Ning Zheng, John D. Scott, Qinghang Liu, William A. Catterall

**Affiliations:** 1 Department of Pharmacology, University of Washington, School of Medicine, Seattle, Washington, USA; 2 Department of Neurobiology and Biophysics, University of Washington, School of Medicine, Seattle, Washington, USA; 3 Howard Hughes Medical Institute, University of Washington, School of Medicine, Seattle, Washington, USA

**Keywords:** voltage-gated Ca^2+^ channels, β-adrenergic stimulation, PKA, Rad, AKAP

## Abstract

Adrenergic modulation of voltage gated Ca^2+^ currents is a context specific process. In the heart Ca_v_1.2 channels initiate excitation–contraction coupling. This requires PKA phosphorylation of the small GTPase Rad (Ras associated with diabetes) and involves direct phosphorylation of the Ca_v_1.2 α_1_ subunit at Ser1700. A contributing factor is the proximity of PKA to the channel through association with A-kinase anchoring proteins (AKAPs). Disruption of PKA anchoring by the disruptor peptide AKAP-*IS* prevents upregulation of Ca_v_1.2 currents in tsA-201 cells. Biochemical analyses demonstrate that Rad does not function as an AKAP. Electrophysiological recording shows that channel mutants lacking phosphorylation sites (Ca_v_1.2 STAA) lose responsivity to the second messenger cAMP. Measurements in cardiomyocytes isolated from Rad^−/−^ mice show that adrenergic activation of Ca_v_1.2 is attenuated but not completely abolished. Whole animal electrocardiography studies reveal that cardiac selective Rad KO mice exhibited higher baseline left ventricular ejection fraction, greater fractional shortening, and increased heart rate as compared to control animals. Yet, each parameter of cardiac function was slightly elevated when Rad^−/−^ mice were treated with the adrenergic agonist isoproterenol. Thus, phosphorylation of Ca_v_1.2 and dissociation of phospho-Rad from the channel are local cAMP responsive events that act in concert to enhance L-type calcium currents. This convergence of local PKA regulatory events at the cardiac L-type calcium channel may permit maximal β-adrenergic influence on the fight-or-flight response.

Acute stress or prolonged exercise evokes β-adrenergic receptor stimulation to enhance cardiac activity (1). In ventricular cardiomyocytes, an early phase of this response involves activation of Ca_v_1.2 channels that conduct L-type Ca^2+^ currents (2, 3, 4). This Ca^2+^ influx initiates excitation–contraction coupling and contributes to the plateau phase of cardiac action potentials, giving time for proximal cardiomyocytes to depolarize (4). L-type calcium channel complexes are composed of five distinct subunits. The α_1_ subunit is the principal functional component. It is composed of four homologous domains (I–IV), each containing six transmembrane segments (S1–S6).

A voltage sensor is formed by the positively charged arginine and lysine residues in the S4 segments. The α_2_, δ, and β subunits modulate ion trafficking and biophysical properties of the pore forming α_1_ subunit (2, 3, 4). Rapid regulation of Ca_v_1.2 currents can proceed through a variety of mechanisms. Originally direct PKA phosphorylation of sites in the cytoplasmic tail of Ca_v_1.2 isoforms was considered the sole means of channel activation (5, 6, 7, 8, 9). However, acute fine-tuning of cardiac L-type Ca^2+^ currents not only involve the action of protein kinases, but importantly requires small GTPases, and ancillary proteins such as A-kinase anchoring proteins (AKAPs) (10, 11, 12, 13, 14, 15).

Members of the RGK GTPase family (Rem, Ras associated with diabetes [Rad], Rem2, GEM/Kir) are potent inhibitors of L-type calcium currents in the heart and neurons (16, 17). Notably, Rad is a principal inhibitor of Ca_v_1.2 (12, 13, 18). Cardiac selective deletion of Rad in mice results in a persistent increase of inotropy without structural or functional remodeling of the heart (12). Rad inhibition can be reversed by PKA phosphorylation which disrupts its interaction between β subunits of the channel (14, 18, 19). Other modes of regulation also contribute to control of Ca_v_1.2 channels. Proteolytic processing of the Ca_v_1.2 α_1_ subunits liberates a C-terminal (CT) domain that serves as an autoinhibitory region (6, 7, 18, 20). Deletion of CT domain in mice reduces Ca^2+^ currents and decreases cell-surface expression of Ca_v_1.2 protein. PKA regulation of truncated Ca_v_1.2 channels is lost (21). We have shown that PKA phosphorylation of Ser1700 contributes to basal and β-adrenergic stimulation of Ca_v_1.2 channel activities (22, 23). Thus, there appears to be multiple means of Ca_v_1.2 regulation (Fig. 1*A*). With this in mind, we explored molecular mechanisms that could act synergistically, or in parallel, to provide convergent adrenergic regulation of Ca_v_1.2 currents.

**Figure 1:**
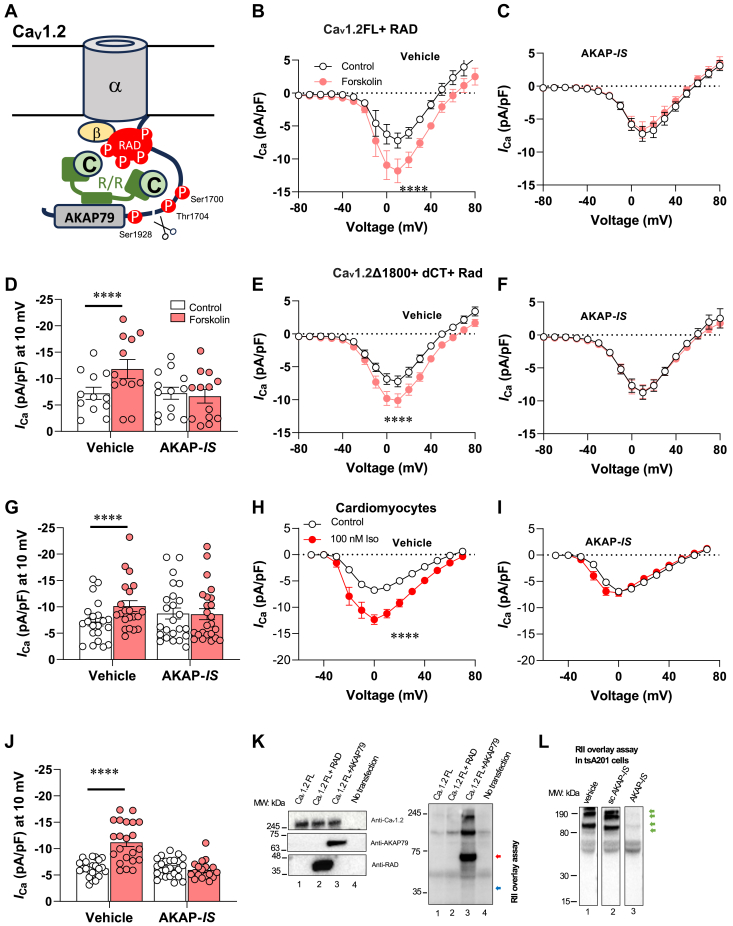
**PKA anchoring contributes to adrenergic modulation of Ca**_**v**_**1.2.***A*, schematic of anchored PKA modulation of Cav 1.2 channels. AKAP79 orients PKA toward phosphorylation sites in the cytoplasmic tail of the channel and the small GTPase Rad. *B*, PKA exists of two type, type I and type II, which are distinguished by their different regulatory subunits RI and RII, respectively. Type II regulatory subunit (RII) is localized *via* interaction with specific AKAPs. An RII overlay assay was performed to investigate the ability of AKAP to bind RIIα of PKA. Characterization of anchoring disruptor peptides were conducted by RII overlay assay of tsA201 cell extracts immobilized on polyvinylidene fluoride membranes. Individual membranes were incubated with RII and vehicle (1), scrambled AKAP-*IS* peptide control (2), or AKAP-*IS* peptide (3). Immunoblot detection of immobilized RII revealed AKAP’s. *Green arrows* present RII binding proteins. *C–K*, AKAP-*IS* eliminates upregulation of Ca_v_1.2 activity by PKA activation in the presence of RAD. Whole-cell patch-clamp recording techniques were used to measure the effects of vehicle (*C*, *F*, and *I*) and 1 μM AKAP-*IS* (*D*, *G*, and *J*) perfused in intracellular solution. I/V curves of Ca_v_1.2 FL of vehicle (*C*) and AKAP-*IS* (*D*) in presence of forskolin (*red*) and vehicle (*white*) and RAD. *E*, statistical analysis peak current induced by depolarizing potential 10 mV in absence (*white*) or presence (*red*) of forskolin. I/V curves of Ca_v_1.2Δ1800 +DCT of vehicle (*F*) and AKAP-*IS* (*G*) in presence of forskolin (*red*) or vehicle (*white*) in presence of RAD. *H*, summarized data on peak current induced by depolarizing potential 10 mV in presence (*red*) or absence (*white*) of forskolin. I/V curves of cardiomyocytes of vehicle (*I*) and AKAP-*IS* (*J*) in presence of isoproterenol (*red*) or buffer control (*white*) in presence of RAD. *K*, summarized data on peak current induced by depolarizing potential 10 mV in presence (*red*) and absence (*white*) of Iso (*G*) and (*H*). Ca_v_1.2 FL + vehicle, N = 12; Ca_v_1.2 FL + AKAP-*IS*, N = 13; Ca_v_1.2Δ1800 +DCT, N = 21; Ca_v_1.2Δ1800 +DCT + AKAP-*IS*, N = 24. In cardiomyocytes, vehicle group with (N = 23) or without Iso (N = 22); AKAP-*IS* group with (N = 24) or without Iso (N = 18). Statistical significance determined *via* Holm–Sidak test following the two-way ANOVA. ∗∗∗∗*p* < 0.0001 *versus* control group. *L*, Ca_v_1.2FL complexes were probed with (*top*) anti-Ca_v_1.2, (*upper mid*) anti-AKAP79, (*lower mid*) anti-RAD antibodies, and (*bottom*) RII overlay, respectively. AKAP, A-kinase anchoring protein; dCT, distal C terminal; Rad, Ras associated with diabetes.

Herein, we show that disruption of PKA–AKAP interactions *via* perfusion of anchoring disruptor peptides (AKAP-*IS*) eliminates upregulation of Ca_v_1.2 activity. This occurs even upon supraphysiological stimulation of cAMP production. Channel mutants lacking Ser1700 and Thr1704 were less regulated by Rad suggesting that phosphorylation at these sites contributes to adrenergic modulation of Ca_v_1.2 currents. In Rad^−/−^ mice, basal Ca^2+^ currents were increased without alteration of expression of Ca_v_1.2 channels. Whole animal analysis by echocardiography shows that Rad KO mice exhibit high basal inotropic responses and a smaller increment in fraction shortening, ejection fraction (EF), and heart rate than WT mice. Yet, isolated Rad^−/−^ ventricular cardiomyocytes still respond to adrenergic stimulation indicating that phosphorylation of Ca_v_1.2 also contributes to channel activation. We provide evidence for AKAPs to synchronize PKA phosphorylation events during adrenergic modulation of Ca_v_1.2 in the cytoplasmic tail of the channel, and on Rad (Fig. 1*A*).

## Results

### PKA anchoring is required for activation of Ca_v_1.2 channels

A defining feature of AKAPs is an amphipathic helix on the surface of the anchoring protein that binds with high affinity to a docking and dimerization domain on regulatory subunits of the kinase (24, 25). Peptide mimetics of this “structural motif” are effective anchoring disruptors and have become the gold standard to confirm the role of anchored PKA in cAMP responsive processes (26, 27, 28). AKAP-*IS* is a potent antagonist peptide that was used to investigate the role of local kinase activity in the stimulation of Ca_v_1.2 currents (29). Cell lysates prepared from tsA201 cells were treated with the AKAP-*IS* or a scrambled peptide control (both 20 μM, Fig. 1*B*). Overlay analyses confirmed that active AKAP-*IS* was an effective antagonist of RII-AKAP interactions for use in cell based electrophysiological experiments (Fig. 1*L*, lane 2).

After transfection for 48 h with channel subunits, Ba^2+^ currents were measured in tsA201 cells. Current-voltage relationships were determined in whole-cell voltage clamp experiments during repeated depolarizations at 10 mV intervals from a resting membrane potential of −80 mV. The amplitude of Ba^2+^ currents was divided by cell capacitance as current density. When 10 μmol/L of the adenylyl-cyclase agonist forskolin (FSK) was added to the extracellular solution, Ca_v_1.2 FL was stimulated at 10 mV when coexpressed with Rad (−FSK: −7.2 ± 1.1 pA/pF, n = 7; +FSK: −11.8 ± 1.8 pA/pF, n = 7; *p* < 0.0001) (Fig. 1, *C* and *E*). A role for PKA anchoring in this process was confirmed upon perfusion of AKAP-*IS*. When the cells were perfused with 1 μmol/L AKAP-*IS*, application of FSK (10 μM) was unable to up-regulate Ba^2+^ currents in Ca_v_1.2 FL (−FSK: −6.9 ± 1.2 pA/pF, n = 13; +FSK: −6.3 ± 1.3 pA/pF, n = 13; *p* = 0.92) (Fig. 1, *D* and *E*). Control studies confirmed that scrambled AKAP-*IS* peptide did not impact cAMP responsive upregulation of Ca_V_1.2 currents (−FSK: −3.9 ± 0.6 pA/pF, n = 18; +FSK: −5.5 ± 1.0 pA/pF, n = 18; *p* < 0.0001) (Fig. S1*A*). Likewise, Ba^2+^ currents increase upon stimulation of cAMP synthesis when the Ca_v_1.2 Δ1800 truncation mutant was coexpressed with the distal C terminal of the Ca_v_1.2 channel protein (−FSK: −7.2 ± 0.8 pA/pF, n = 21; +FSK: −10.1 ± 1.0 pA/pF, n = 21; *p* < 0.0001) (Fig. 1, *F* and *H*). As expected, cAMP responsive stimulation of the reconstituted channel complex was sensitive to AKAP-*IS* (−FSK: −8.8 ± 1.1 pA/pF, n = 22; +FSK: −8.7 ± 1.1 pA/pF, n = 22; *p* = 0.99) (Fig. 1, *G* and *H*). Additional controls confirmed that scrambled AKAP-*IS* had no effect on the reconstituted Ca_v_1.2 Δ1800 with the heterologous expressed CT domain (−FSK: −3.1 ± 0.6 pA/pF, n = 18; +FSK: −3.9 ± 0.9 pA/pF, n = 12; *p* < 0.0001) (Fig. S1*B*).

To test this phenomenon in a more physiological context, isolated mouse cardiomyocytes were subjected to β-adrenergic activation with isoproterenol (Iso). As expected, the L-type Ca^2+^ current increased significantly (−Iso: −6.2 ± 0.3 pA/pF, n = 23; +Iso: −11.2 ± 0.8 pA/pF, n = 22; *p* < 0.0001) (Fig. 1, *I* and *K*). Importantly application AKAP-*IS* eliminated upregulation of L-type Ca^2+^ currents (−Iso: −6.4 ± 0.4 pA/pF, n = 23; +Iso: −6.0 ± 0.4 pA/pF, n = 18; *p* = 0.97) (Fig. 1, *J* and *K*). Collectively these electrophysiology results further argue that disruption of PKA interaction with AKAPs prevents upregulation of Ca_v_1.2 currents.

A logical next step was to investigate if the channel-associated protein Rad could function as an AKAP. To test this idea, tsA-201 cells were transfected with complementary DNAs encoding Ca_v_1.2. Coexpression of Rad or the channel associated AKAP79 provided putative PKA-anchoring proteins. Ca_v_1.2 immune complexes were probed by RII overlay assay (11). A band corresponding to AKAP79 was detected by RII overlay (Fig. 1*L*, lane 3). No signal corresponding to Rad was detected (Fig. 1*L* lane 2). Thus, we conclude that Rad is not an AKAP that sequesters PKA to Ca_v_1.2 channels.

### PKA phosphosites on Rad and the Ca_v_1.2 α_1_ subunit mediate channel regulation

**Figure 2:**
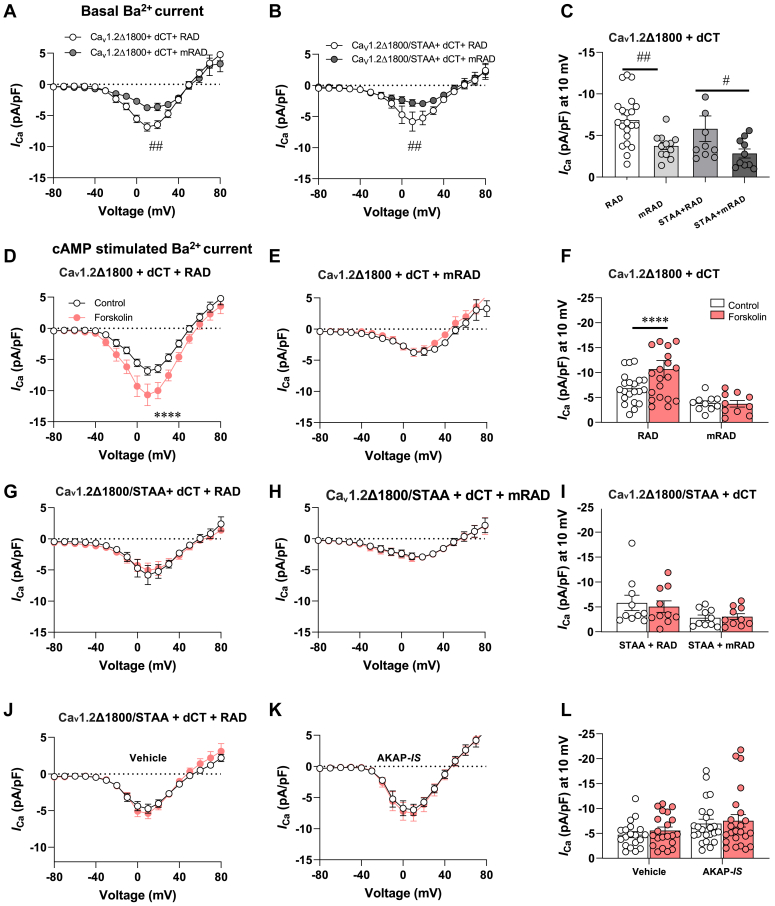
**Phosphosites on Ca**_**v**_**1.2 and Rad are necessary for cAMP stimulation of currents.***A–C*, basal electrophysiological properties of truncated (*A*) Ca_v_1.2 and (*B*) the STAA nonphosphorylatable mutant were assessed. Recordings were conducted in the presence of Rad (*open circles*) or the mRad (*gray circles*) mutant that cannot be phosphorylated by PKA. *C*, summarized data on peak current induced by depolarizing potential 10 mV in (*A*) and (*B*). *D–F*, I/V curves of Ca_v_1.2Δ1800 + dCT coexpressed with RAD WT (*D*) and m RAD (*E*) in presence (*red*) or absence (*white*) of forskolin. *F*, summarized data on peak current induced by depolarizing potential 10 mV in (*D*) and (*E*). *G–I*, I/V curves of (*G*) Ca_v_1.2Δ1800/STAA + dCT coexpressed with RAD WT and (*H*) mRAD (*H*) with phosphoregulatory in presence (*red*) or absence (*white*) of forskolin. *I*, summarized data on peak current induced by depolarizing potential 10 mV in (*G*) and (*H*). *J* and *K*, I/V curves of vehicle (*J*) and AKAP-*IS* (*K*) in presence of RAD. *L*, summarized data on peak current induced by depolarizing potential 10 mV in presence (*red*) and absence (*white*) of forskolin (*J*) and (*K*). Ca_v_1.2Δ1800/STAA + vehicle, N = 19; Ca_v_1.2Δ1800/STAA + scramble AKAP-*IS* N = 18; Ca_v_1.2Δ1800/STAA + AKAP-*IS*, N = 23, Statistical significance determined *via* Holm–Sidak test following the two-way ANOVA. ^#^*p* < 0.05 and ^##^*p* < 0.01 *versus* WT RAD; ∗∗∗∗*p* < 0.0001 *versus* control group. AKAP, A-kinase anchoring protein; dCT, distal C terminal; Rad, Ras associated with diabetes.

Our previous studies show that mutant Ca_v_1.2 channels lacking phosphoregulatory sites at Ser1700 and Thr1704 exhibit low basal currents in heterologous systems and heart cells (16). Rad is also a PKA substrate and phosphorylation at multiple sites contributes to cAMP responsive derepression of Ca_v_1.2 currents (12, 13, 14). This information has allowed the generation of a nonactivatable mutant form of Rad where PKA phosphorylation sites are changed to alanines (Ser25Ala, Ser38Ala, Ser272Ala, and Ser300Ala (14)). Initially, we tested the role of Rad and its nonactivatable ortholog in the regulation of basal Ca_v_1.2 channel activities. There was a reduced effect on basal channel activity when either Rad or mRad was expressed with Ca_v_1.2Δ1800 (Rad: −6.8 ± 0.6 pA/pF, n = 21; mRad: −3.7 ± 0.4 pA/pF, n = 12; *p* = 0.0068) (Fig. 2, *A* and *C*). Likewise, Rad modulation of basal channel activity was minimal when these experiments were repeated in cells expressing the Ca_v_1.2Δ1800/STAA mutant and the free CT regulatory domain (Rad: −5.2 ± 1.5 pA/pF, n = 10; mRad: −2.8 ± 0.5 pA/pF, n = 10; *p* = 0.011) (Fig. 2, *B* and *C*).

Additional studies evaluated the impact of Rad forms under conditions of maximal cAMP stimulation. When the cells were treated with 10 μmol/L FSK, the peak Ba^2+^ currents were increased in Ca_v_1.2 Δ1800 with WT Rad (−FSK: −6.8 ± 0.6 pA/pF, n = 21; +FSK: −10.7 ± 1.7 pA/pF, n = 20; *p* = 0.0015) (Fig. 2, *D* and *F*). There was no change in Ca_v_1.2Δ1800 with the mRad condition (−FSK: −3.7 ± 0.4 pA/pF, n = 12; +FSK: −3.7 ± 0.6 pA/pF, n = 22; *p* = 0.99) (Fig. 2, *E* and *F*). There was no cAMP response in the presence or absence of Rad orthologs when these studies were repeated using the Ca_v_1.2 Δ1800/STAA channel mutant (−FSK: −5.8 ± 1.5 pA/pF, n = 10; +FSK: −5.0 ± 1.2 pA/pF, n = 10; *p* = 0.99) and mRad (−FSK: −2.8 ± 0.5 pA/pF, n = 10; +FSK: −3.0 ± 0.6 pA/pF, n = 10; *p* = 0.99) (Fig. 2, *G–I*). Further controls confirmed that AKAP-*IS* mediated disruption of PKA anchoring had no impact on Ca_v_1.2 currents in the presence of mutant Rad and when the Ser1700 and Thr1704 phosphorylated sites were mutated (−FSK: −6.9 ± 0.9 pA/pF, n = 23; +FSK: −7.5 ± 1.3 pA/pF, n = 23; *p* = 0.99) (Fig. 2, *J–L*). Together, these results confirm that phosphorylation of Rad modulates adrenergic stimulation of Ca_v_1.2 and that Ser1700 on the channel protein also contribute to activation.

### Rad deficiency stimulates basal inotropy and impairs the β-adrenergic response

**Figure 3:**
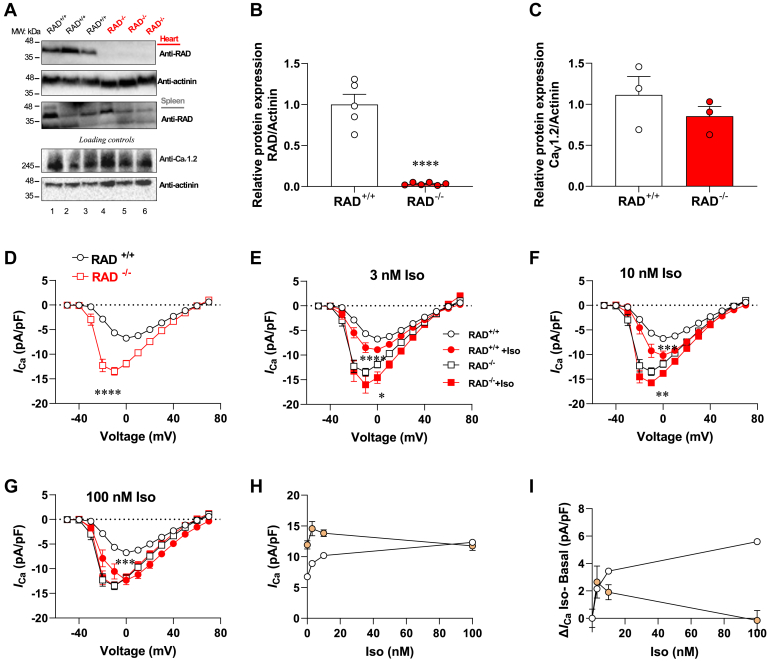
**Altered basal and β-adrenergic stimulation of L-type Ca**^**2+**^**currents in adult RAD deficiency ventricular cardiomyocytes.***A*, immunoblot analysis of RAD expression in (*top*) cardiac and (*middle*) spleen lysates from control (RAD^+/+^, *black*, lanes 1-3) and cardiac selective (RAD^−/−^, *red*, lanes 4-6) KO mice. Immunoblot detection of actinin and Ca_v_1.2 (4 weeks posttamoxifen treatment) serve as loading controls Quantification by densitometry of (*B*) RAD levels and Ca_v_1.2 a subunit (*C*) in the heart tissues. Statistical significance determined *via* unpaired *t* test, ∗∗∗∗*p* < 0.0001 *versus* RAD^+/+^ mice. *D*, I/V curves of basal L-type Ca^2+^ current in RAD^+/+^ (n = 23) and RAD^−/−^ ventricular cardiomyocytes (n = 12). *E–G*, I/V curves of L-type Ca^2+^ current in absence and presence of Iso at 3 nM (*E*), 10 nM (*F*), and 100 nM (*G*). *H*, peak current density at 0, 3, 10, and 100 nM Iso in RAD^+/+^ and RAD^−/−^ ventricular cardiomyocytes. *I*, concentration–response curve of Iso-elicited increase in current (baseline subtracted). Statistical significance determined *via* Holm–Sidak test following the two-way ANOVA. ∗*p* < 0.05, ∗∗*p* < 0.01, ∗∗∗*p* < 0.001, and ∗∗∗∗*p* < 0.0001 *versus* groups without Iso. Iso, isoproterenol; Rad, Ras associated with diabetes.

Phosphorylation of Rad is a critical element in the Ca_v_1.2 activation pathway. To further investigate this interesting means of convergent regulation, we produced mice with a cardiac specific deletion of Rad. Immunoblot analysis of tissue lysates show that Rad protein is ablated from the heart but expressed in spleen (Fig. 3, *A* and *B*).

Various studies have used high doses of Iso for β-adrenergic stimulation of Ca_v_1.2 in transgenic mouse models (Table S1; (12, 14, 18, 30)). Therefore, we tested the effect of channel modulation by physiologic levels of Iso. Initially, we measured whole-cell Ca^2+^ currents in adult cardiomyocytes at basal level and upon perfusion of Iso over a range of concentrations (3, 10, and 100 nM Iso). The basal L-type Ca^2+^ current was markedly increased in Rad^−/−^ cardiomyocytes compared that of Rad^+/+^ cardiomyocytes (Rad^+/+^: −6.7 ± 0.4 pA/pF, n = 23; Rad^−/−^: −11.9 ± 0.6 pA/pF. pA/pF, n = 12; *p* < 0.0001) (Fig. 3*D*). There was no alteration in the level of channel protein (Fig. 3, *A* and *C*). The half activation voltage V_1/2_ was shifted toward more negative membrane potential (Rad^+/+^: −14.2 ± 1.3 pA/pF, n = 23; Rad^−/−^: −24.5 ± 1.3 pA/pF, n = 12; *p* < 0.0001) (Fig. S2*A*). The steady-state inactivation had no difference of V_1/2_ between Rad^+/+^ and Rad^−/−^ (Rad^+/+^: −19.5 ± 1.0 pA/pF, n = 23; Rad^−/−^: −22.0 ± 1.2 pA/pF, n = 12; *p* = 0.14) (Fig. S2*B*). Treatment of Rad^+/+^ ventricular cardiomyocytes with 3 nM Iso increased the peak L-type Ca^2+^ current (−Iso: −6.7 ± 0.4 pA/pF; +Iso: −8.9 ± 0.6 pA/pF) (Fig. 3*E*). In contrast, there was an incremental increase of L-type Ca^2+^ current in Rad^−/−^cardiomyocytes. (−Iso: −11.9 ± 0.6 pA/pF; +Iso: −14.6 ± 1.1 pA/pF) (Fig. 3*E*).

When using 10 nM Iso, we noted that the peak L-type Ca^2+^ current was increased by −10.2 ± 0.9 pA/pF in Rad^+/+^ cardiomyocytes (Fig. 3*F*). There was a proportional incremental increase of L-type Ca^2+^ current by −13.8 ± 0.5 pA/pF in Rad^−/−^ ventricular cardiomyocytes (Fig. 3*F*). At the highest concentration of agonist (100 nM) the L-type Ca^2+^ current was increased by −12.3 ± 0.9 pA/pF in Rad^+/+^ cardiomyocytes (Fig. 3*G*). Interestingly at this saturating concentration of Iso, no change was observed in Rad^−/−^ cardiomyocytes (Fig. 3*G*). Cardiomyocytes of both genotypes have the same level of L-type calcium channels. The full impact of the Rad deficiency is more fully illustrated by dose–response curves that plot the absolute magnitude of the L-type current *versus* Iso concentration (Fig. 3*H*). Iso dose responsive effects are evident in these concentration–response curves. *i*) Basal L-type calcium channels have higher basal activity in Rad^+/+^ cardiomyocytes. *ii*) In Rad^+/+^ cardiomyocytes, there is a modest incremental increase of L-type Ca^2+^ current upon low stimulation with Iso (3 and 10 nM). *iii*) At saturating concentrations, no response to Iso is detectable in Rad^−/−^cardiomyocytes. We plotted the basal-subtracted incremental increase in pA/pF *versus* Iso concentration showing that PKA-dependent upregulation of Ca_v_1.2 activity is altered in Rad KO ventricular cardiomyocytes (Fig. 3*I*). Thus, cardiac deletion of Rad promotes high basal L-type Ca^2+^ current and impairs the β-adrenergic response.

**Figure 4:**
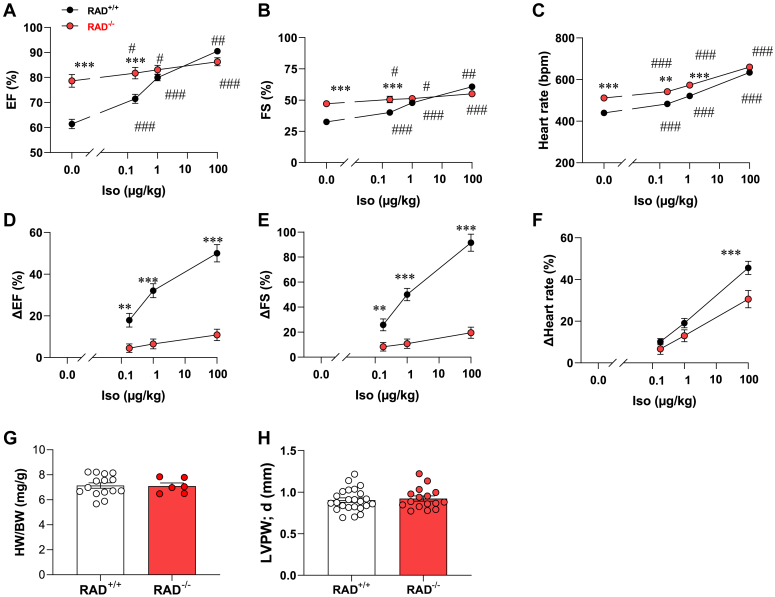
**Inotropic dose response to β-adrenergic stimulation in RAD**^**−/−**^**mice.** Isoproterenol dose response assessed (*A*) ejection fraction, (*B*) fractional shortening, and (*C*) heart rate in RAD^+/+^ (*black*; n = 24) and RAD^−/−^ mice (*red*; n = 18) mice. Isoproterenol administration was by intraperitoneal injection. *D* and *E*, isoproterenol dose responsive changes (*D*) ejection fraction, (*E*) fractional shortening and (*F*) heart rate are indicated for RAD^+/+^ (*black*; n = 24) and RAD^−/−^(*red*; n = 18) mice. Summarized data of (*G*) the ratio of heart weight to body weight and in RAD^+/+^ (*white*, n = 15) and RAD^−/−^ (*red*; n = 6) mice. *H*, summarized data on left ventricular posterior wall in RAD^+/+^ (*white*; n = 24) and RAD^−/−^ mice (*red*; n = 18) mice. Statistical significance determined *via* Holm–Sidak test following the two-way ANOVA. ∗∗*p* < 0.01 and ∗∗∗*p* < 0.001 *versus* RAD^+/+^ mice; ^#^*p* < 0.05 and ^##^*p* < 0.01 *versus* saline injection. Rad, Ras associated with diabetes.

Next, we measured cAMP responsive regulation of cardiac contractility and function *in vivo* by echocardiography. Three cardiac parameters have been measured. First, cardiac selective Rad KO mice exhibited higher baseline left ventricular EF (Rad^+/+^: 61.4% ± 1.8%, n = 24; Rad^−/−^: 78.6% ± 2.5%, n = 16; *p* < 0.0001) (Fig. 4*A*). This is a measurement of how much blood the left ventricle pumps out with each contraction of the heart. Second, FS, a measurement of ventricular contractility, was greater in the Rad^−/−^ cohort than control mice (Rad^+/+^: 32.5% ± 1.2%, n = 24; Rad^−/−^: 47% ± 2.5%, n = 16; *p* < 0.0001) (Fig. 4*B*). Third, the heart rate of Rad KO mice was 511.2 ± 13.3 beats per min (n = 16) as compared to 439.5 ± 8.9 beats per min (n = 24) in control animals (*p* < 0.0001 Fig. 4*C*).

Circulating plasma levels of the hormone epinephrine range from 60 to 180 ng/kg during an adrenal response in mice (31, 32). Therefore, we compared *in vivo* inotropic responses of mice at physiologic doses of Iso (0.25 μg/kg and 1 μg/kg). To evoke supraphysiological responses, echocardiography measurements were also conducted at 100 μg/kg of Iso. Iso dosing enhanced each parameter of cardiac function more effectively in control animals than Rad^−/−^ mice (Fig. 4, *A–C*). Yet, in Rad^−/−^ mice cardiac parameters were slightly but significantly elevated in response to each concentration of Iso (Fig. 4, *A–C*). These differences are more evident in Figure 4, *D–F* that plot dose responsive changes in cardiac output. Iso-induced changes in left ventricular EF are modest in Rad^−/−^ mice (Fig. 4*D*, red) but significantly more pronounced in floxed homozygote control animals (Fig. 4*D*, black). A similar trend is observed when rates of changes for fractional shortening and heart rate are plotted (Fig. 4, *E* and *F*). Control experiments confirmed that the ratio of heart weight to body weight (Fig. 4*G*) and the thickness of left ventricular posterior wall (Fig. 4*H*) were the same in both genotypes. Collectively, these data maintain that factors other than Rad contribute to adrenergic stimulation of the heart. Electrophysiological evidence indicates that PKA phosphorylation of Ca_v_1.2 is a contributing factor.

## Discussion

The heart is innervated by the sympathetic nervous system and responds to adrenergic hormones, particularly during episodes of acute stress. The intracellular influence of epinephrine and norepinephrine proceeds through mobilization of the second messenger cAMP to activate PKA (1). Kinase modulation of L-type Ca^2+^ currents increases contractility and cardiac performance (1). This report investigates two targets that are coincidently or coordinately phosphorylated by PKA, the small GTPase Rad, and the Ca_v_1.2 α_1_ subunit.

Modulation of Ca_v_1.2 by Rem and Rad small GTPases is a principal means of unleashing the adrenergic response in cardiomyocytes (33). Elegant studies by Marx and colleagues argue that PKA-dependent repression of Rad is a key mode of Ca_v_1.2 channel regulation in the heart (18, 19, 33). Results presented in Figures 2 and 3 of this study infer that PKA phosphorylation of sites in the CT tail of the channel may further contribute to this process. This is supported by evidence that substitution of alanine at Ser1700 and Thr1704 tempers beta-adrenergic Ca_v_1.2 currents in ventricular myocytes (22). Although some question if these phosphorylation events contribute to full adrenergic stimulation, these and other PKA phosphosites are operational in the modulation of Ca_v_1.2 in smooth muscle (34, 35).

Several factors may explain these conflicting results. There may be topological distinctions in the conformation of full-length Ca_v_1.2 as compared to the reconstituted Ca_v_1.2 Δ1800 + distal C terminal regulatory tail used in aspects of our study. Also, the Marx study introduced 35 mutations of the channel to eliminate all phospho sites (18). While there are merits to this comprehensive approach, processive substitution of so many serine’s and threonine’s may have an unanticipated structural impact on channel regulatory events. Additionally, different genetic strategies generated the mouse models used in each study. This may have inadvertently altered the electrogenic properties of Ca_v_1.2. Likewise, unreported variation in experimental conditions and cell types used in each study might trigger differential effects on channel modulation. It is also known that Ca_v_1.2 current is increased when Rad is knocked out (12). Interestingly, the original reports only tested supraphysiological doses of β-adrenergic Iso. Therefore, we considered it important to evaluate the effect of Iso at physiologically relevant levels of β-adrenergic stimulation in Rad-deficient mice (Fig. 3, *E–G*). Thus, adrenergic modulation of cardiac L-type calcium channels may be a complex and context-specific process.

The findings extend our knowledge of Ca_v_1.2 channel regulation in four ways. First, we use anchoring disruptor peptides to confirm that local PKA activity abrogates adrenergic modulation of Ca_v_1.2 currents (29). These experiments add to a growing body of data implicating AKAPs as positional modulators of PKA phosphorylation of channel complexes (36). AKAP79/150 was the first anchoring protein linked to cAMP responsive potentiation of Ca_v_1.2 (37, 38). Subsequent studies have also implicated the AKAP15/18 gene family (39, 40), and Cypher/Zasp (41) as alternate channel-associated anchoring proteins. AKAP79/150 and AKAP15/18 are tethered to the cytoplasmic tail of Ca_v_1.2 *via* conserved leucine-zipper motif, thereby maintaining PKA holoenzymes within a few microns of their target substrates (42, 43). AKAP79/150 can form dimers and has been implicated in clustering of Ca_v_1.2 channels to create zones of maximal calcium entry (44, 45). Consequently, it is unlikely that any individual AKAP dominates in the spatial control of Ca_v_1.2 modulation, particularly as adrenergic stimulation of this channel is retained in adult cardiomyocytes isolated from AKAP150^−/−^, AKAP18^−/−^, and double KO mice (46, 47, 48). Such redundancy in AKAP-PKA control of Ca_v_1.2 activation infers that this signaling step is an essential cardiac regulatory event (35).

Phosphorylation of Rad is required for dissociation of the Rad–β_2_b interaction and β-adrenergic stimulation mediated activation of Ca_v_1.2 channel (18). PKA anchored directly to the α1 subunit of Ca_v_1.2 channels by AKAPs is necessary for reconstitution of PKA-dependent modulation of the channel in heterologous express systems and in cardiomyocytes (49). Although we empirically show that Rad is not an AKAP, we cannot exclude the possibility that Rad functions to bridge other anchored PKA complexes to the channel.

Second, some of these Ras-like effectors are PKA-anchoring proteins (50). Yet, the biochemical analyses presented in Figure 1 exclude the possibility of Rad functioning as an AKAP (50, 51, 52). Standard diagnostic tests for AKAPs such as RII overlay and coprecipitation of PKA subunits are negative (49). Rad was originally identified as a gene upregulated in patients with type 2 diabetes (16). Subsequent cell-based studies established that Rad and certain paralogs profoundly inhibit Ca_v_1/Ca_v_2 trafficking and channel activity (12, 13, 18). Rad suppression of Ca_v_1.2 action is mediated by direct binding of the GTPase to the cytoplasmic face of the β subunit of the channel complex as shown in Figure 1*A*. Elegant studies have established that PKA phosphorylation at multiple sites in Rad promotes dissociation from Ca_v_1.2 to enhance calcium conductance (14). This asserts that Rad is optimally positioned to restrain Ca_v_1.2 and has preferred access to PKA. These ties nicely with recent evidence that active, intact, and anchored PKA holoenzymes are sequestered within 2 to 4 cubic microns of target substrates (42, 53, 54, 55, 56). Under this scenario, AKAP79/150 or AKAP15/18 associated pools of PKA would be equally well positioned to phosphorylate Rad and sites within the CT tail of Ca_v_1.2.

This latter postulate leads credence to our third conclusion presented in Figure 2, *G–L*. We show in Figure 2, Ca_v_1.2 variants with phosphoregulatory mutations in the cytoplasmic CT domain of the channel are not responsive to cAMP. Importantly the mutant channel is refractory to *i*) supraphysiological stimulation of cAMP synthesis with the diterpene FSK, *ii*) nonresponsive to Rad, and *iii*) peptide disruption of PKA–AKAP interactions. One caveat of our result is that all experiments were performed upon truncated forms of Ca_v_1.2 and in the presence of the free cytoplasmic (CT) tail of the channel. Thus, we cannot eliminate the possibility that excess CT tail could serve as a buffer to requisition and redirect Rad and AKAPs away from their sites of local action. Nonetheless, an important conclusion from these studies is that direct PKA phosphorylation of the channel may contribute to changes in the electrogenic properties of Ca_v_1.2.

There are other factors to consider as we ponder the implications of this latter regulatory mechanism. The Ser 1700 and Ser 1928 sites used in neuronal and smooth muscle L-type calcium channels are consensus PKA motifs (5, 34). These two regions are highly conserved (Fig. S3). Several groups have shown that these side chains are rapidly phosphorylated upon adrenergic stimulation of channel associated pools of PKA. Hence, it is likely that Ca_v_1.2 phosphorylation will be completed before the multisite phosphorylation of Rad is accomplished. This latter signaling event is necessary for its release from the vicinity of the channel. Future studies will be directed toward defining if PKA phosphorylation of Ca_v_1.2 and Rad is sequential or stochastic. It is also important to note that anchoring proteins such as AKAP79/150 and Cypher/Zasp constrain multivalent scaffolds that include kinases and the Ca^2+^/calmodulin-dependent phosphatase calcineurin/PP2B (41, 57, 58, 59, 60). Such anchored phosphatase configurations could provide the means for local signal termination of Ca_v_1.2 currents *via* dephosphorylation of Rad and channel subunits. The Thr 1704 site on Ca_v_1.2 is a target for CK2 (61). This kinase is not responsive to cAMP and its mode of activation is enigmatic. Nevertheless, phosphorylation of CK2 sites is often favored by the presence of a priming phosphate 3 to 4 residue upstream of the target motif (62, 63).

Intriguingly, pSer1700 is optimally positioned to fulfill this role and covalent modification of this site may be a necessary prelude to CK2 phosphorylation of Thr 1704. This hitherto unrecognized layer of channel regulation may occur as a secondary consequence of adrenergic stimulation. Furthermore, Glu47 on junctophilin-2 is involved in ionic interactions with Arg1599 on Ca_v_1.1. This corresponds to Arg1745 in Ca_v_1.2 which is located downstream of these phosphorylated sites (64). Yet, junctophilin-2 KO mice do not exhibit changes in basal L-type calcium currents (65). Thus, it is unlikely that junctophilin binding influences basal phosphorylation of Ser1700/Thr1704.

Fourth, electrophysiological and whole animal studies in Rad^−/−^ mice provide an ideal way to assess the relative contribution of different elements in local PKA activation of Ca_v_1.2. The elevated basal L-type Ca^2+^ currents recorded in cardiomyocytes isolated from Rad^−/−^ mice presented in Figure 3 offer unequivocal evidence of a role for this small GTPase as an essential modulator of Ca_v_1.2 action. Yet, our results show that Rad-deficient mice still exhibit incremental stimulation of channel activity at low physiological doses of the agonist Iso. Future studies will be directed toward resolving mechanisms of convergent regulation by Rad and any additional influence of direct PKA phosphorylation of Ca_v_1.2.

Whole animal echocardiography measurements presented in Figure 4 further support this conclusion showing a 30 to 40% increase in baseline left ventricular EF, FS, and heart rate in cardiac selective Rad KO animals. These observations argue that association with Rad is important to suppress Ca_v_1.2 channels to maintain baseline cardiac function. Although adrenergic stimulation is reduced, it is not completely abolished. These latter findings are perhaps most evident in Figure 4, *D* and *E*, where dose responsive changes to physiologic levels of Iso are plotted against changes in cardiac output. Accordingly, Rad^−/−^ mice retain an element of sensitivity to adrenergic stimulation that could be explained by direct phosphorylation of Ser 1700 by PKA and subsequent modification of Thr 1704.

In conclusion, we propose that adrenergic stimulation of cardiac L-type calcium currents is a multifactorial local cAMP response. The concurrent or coincident phosphorylation of Rad and Ca_v_1.2, and the concomitant release of this GTPase are essential activation events that initiate excitation contraction coupling. New developments in our understanding of cAMP generation and the 3D utilization of this intracellular second messenger have revealed receptor-associated independent cAMP nanodomains that contain all the necessary components to elicit rapid and local PKA signaling (55, 66, 67). Hence the intracellular responses to adrenergic stimulation may be regulated by channel-associated or channel proximal AKAP signaling islands that confer the bidirectional phosphorylation of Rad and Ca_v_1.2 to control these vital calcium currents.

## Experimental procedures

### Cell culture and cell transfection

Human embryonic kidney tsA-201 cells were cultured in Dulbecco's modified Eagle's medium/Ham’s F12 supplemented with 10% fetal bovine serum and 100 units/ml penicillin and streptomycin. Cells were grown ∼70% confluence in 6% CO_2_ and transiently transfected as previously described (68). The constructs of Ca_v_1.2 subunits and Rad used in this study include rabbit Ca_v_1.2 α_1_ (NP_001129994.1), rat β_2_b (XP_006254365.1), rat α_2_δ (NP_001104317.1), Rad (NP_001412596.1). The sequence of the Ca_v_1.2 α_1_ subunit in rabbit (P15381-1), *Homo sapiens* (Q13936-1), rat (P22002), and mouse (Q01815) are aligned in UniProt database.

### Animals

All experimental procedures and protocols were approved by the Animal Care and Use Committee of the University of Washington and conformed to the National Institute of Health “Guide for the Care and Use of Laboratory Animals.” The RAD^−/−^ mice were provided by Dr Satin from University of Kentucky. RAD deficiency was induced as previously described (12). All mice received a single intraperitoneal injection with tamoxifen (40 mg/kg body weight) and then were used over 4 weeks after tamoxifen treatment.

### RII overlay assay and Western blot

Forty-eight hours posttransfection, cells washed in the lysis buffer containing Hepes, 20 mM, 150 mM NaCl, 1 mM sodium orthovanadate, 20 mM NaF, 5 mm EDTA, 1% Triton X-100, plus one tablet protease inhibitors cocktail, phosphatase inhibitor mixture 2 (Sigma, P5726, ×100), and phosphatase inhibitor mixture 3 (Sigma, P0044, ×100) with pH adjusted to 7.4 with NaOH, and then mixed by rotation at 4 °C for 10 min. Unsolubilized material was removed by centrifugation at 14,000 rpm. The supernatants were coimmunoprecipitated with 5 μg anti Ca_v_1.2 antibody (Jackson ImmunoResearch) following the protocol of dynabeads protein G immunoprecipitation kit (Thermo Fisher Scientific). The elute solution was prepared in 4× SDS-PAGE loading buffer resolved on 4 to 12% SDS-PAGE gels and transferred to nitrocellulose membrane. Immunoblotting was performed with the RIIα-His protein (1:2500) and incubated overnight at 4 °C on a rocker. Then incubating anti 6× His horseradish peroxidase antibody at a 1:5000 dilution in 5% milk in tris-buffered saline. Finally, immunoblots were developed and quantified using the Bio-Rad ChemiDoc MP Imaging System. At the same time, the same immunoblots were performed with anti Ca_v_1.2 antibody (1:1000), anti-AKAP79 antibody (1:1000, Cell Signaling Technology), and anti RAD antibody (1:1000, Everest Biotech), respectively.

AKAP-*IS* peptide (QIEYLAKQIVDNAIQQAKA) and scrambled AKAP-*IS* (QDVEIQLKAAYNQKLIAIA) with purity of 98% were synthesized from Bio basic company. The peptides were dissolved in dimethylsulfoxide and stored at −20 °C. The peptides were incubated with RIIα-His protein for 30 min at 4 °C on a rocker and then the mix solution was used for immunoblotting nitrocellulose membrane for RII overlay assay.

Heart and spleen tissues excised from Rad^+/+^ and Rad^−/−^ were flash-frozen, and tissue was pulverized using Freezer Mill before sonication in cell lysis buffer. Lysates were centrifuged at 14,000 rpm, and protein concentrations of supernatants were measured. Lysates were prepared in loading buffer with 60 μg total proteins. Immunoblots were performed with anti Ca_v_1.2 antibody (1:1000), anti Rad antibody (1:1000, Everest Biotech), anti actinin antibody (1:1000, Cell Signaling).

### Electrophysiology

For tsA201 cells, experiments were performed at room temperature (23–25 °C). an Axon 200B patch-clamp amplifier (Axon Instruments) was used for patch clamp. The cells were bathed in solution containing the following (in mmol/l): 150 Tris, 10 BaCl_2_, 1 MgCl_2_,10 glucose with the pH adjusted to 7.4 with MeSO_4_.The intracellular solution contained 135 mM CsCl, 10 mM EGTA, 1 mM MgCl_2_, 4 mM MgATP, and 10 mM Hepes with pH adjusted to 7.3 with CsOH. Currents were stimulated by depolarization at 10 mV intervals ranging 80 mV to + 80 mV using 3 to 5 MΩ glass electrodes with 50 to 70% resistance compensation. Current traces were sampled at 5 kHz after antialias filtering at 2 kHz. Current amplitudes during each depolarization were normalized to cell capacitance. The peptides dimethylsulfoxide stock solution was diluted 5000 times to work concentration of 1 μM in pipette solution. The cells were incubated with peptides for 5 min before patch recording after whole-cell configuration was established. On the day of experiments, the stock solution was diluted to the desired concentrations with the extracellular solution.

Single ventricular myocytes were enzymatically isolated as described previously (16). The cells were bathed in solution containing the following (in mmol/l): 140 NaCl, 1.8 CaCl_2_, 5 CsCl, 1.2 MgCl_2_,10 glucose, 10 Hepes, 4 4-AP with the pH adjusted to 7.4 with NaOH. The intracellular solution contained 135 CsCl, 15 TEA-Cl, 1 MgCl_2_, 5 MgATP, 10 mM BAPTA, and 10 Hepes with pH adjusted to 7.2 with CsOH. Ca^2+^ current was elicited by 1-s depolarizing steps from a holding potential of −70 mV to potentials ranging from −70 mV to +70 mV in 10 mV increments. Pipette resistances were 1.5 to 3 MΩ. Current is expressed as current density (normalized cell capacitance). The activation curve (conductance-voltage relationships) was derived from the current-voltage relationships obtained by plotting the peak current from −70 mV to 0 mV according to:

G=I/(V−Vrev)

where G is the conductance, *I* the recorded peak current at different test potential V, and Vrev the apparent observed Ca^2+^ reversal potential. The voltage-dependent relationships of Ca^2+^ current was fitted by the following Boltzmann equation:

G=Gmax/{1+exp[(V−V1/2)/k]}

where Gmax is the maximal conductance, V_1/2_ the voltage for the half-maximal activation, and k the slope factor. The inactivation relationships were evaluated by a Boltzmann equation fit to the normalized to peak currents from −70 mV to +0 mV as follows:

I/Imax=1/{1+exp[(V−V1/2)/k]}

where the *I/I*max (fraction) was the current at the test potential normalized to the peak current. V_1/2_ the voltage for the half-maximal inactivation and k the slope factor.

### Echocardiography

For echocardiographic analysis, mice were transferred into an anesthetic induction chamber and anaesthetized using 2% (vol/vol) isoflurane mixed with 1 l/min O_2_ by inhalation. Anaesthetized state was maintained outside of the chamber with 2%; isoflurane and 1 l/min 100% O_2_. Body temperature was maintained at 37 °C *via* a heating pad and lamp. M-mode image derived from the short axis of left ventricle were recorded in 3 to 5 cycles using Visual Sonics Vevo 2100 imaging system. After the baseline echocardiographic recording, these mice aged 12 to 16 weeks were received three doses of 0.25 μg/kg, 1 μg/kg, and 100 μg/kg Iso *via* intraperitoneal injection. Echocardiographic data were collected 2 min after drug administration. Left ventricular end-diastolic dimension (LVID;d) and end-systolic dimension (LVID;s) were measured by M-mode tracing. The percentage of FS was calculated as equation: ([LVID;d − LVID;s]/LVID;d) × 100, and EF using the equation: ([EDV−ESV]/EDV) × 100, where EDV represents end-diastolic volume and ESV represents end-systolic volume.

## Statistical analysis

Data are presented as the mean ± SEM, with the SEM indicated by the error bars. Where appropriate, student’s t test, two-way ANOVA with repeated measures was applied to determine statistical differences. *p* < 0.05 was considered statistically significant. All statistical analyses were performed using GraphPad Prism 8.

## Data availability

All data are contained within the article.

## Supporting information

This article contains supporting information.

## Conflict of interest

The authors declare that they have no conflicts of interest with the contents of this article.
