# Supplementary material for: Anchored PKA synchronizes adrenergic phosphoregulation of cardiac Cav1.2 channels
Source: J Biol Chem. 2024 Aug 10;300(9):107656. doi: 10.1016/j.jbc.2024.107656 (PMC11408856; doi:10.1016/j.jbc.2024.107656)
Supplement: Supplemental Figures S1–S3 Legend [file mmc1.docx]

**Supporting Figure 1.** Scramble AKAP-*IS* didn’t affect the increase calcium current induced by forskolin. I/V curves of Ca_v_1.2 FL (A) and Ca_v_1.2Δ1800 +DCT (B) in presence of forskolin (red) and no forskolin (white) in presence of RAD. Ca_v_1.2 FL + scramble AKAP-*IS*, N=18; Ca_v_1.2Δ1800 +DCT + scramble AKAP-*IS*, N=12; Statistical significance determined via Holm-Sidak test following the Two-Way ANOVA. *****P*<0.0001 vs control group.

**Supporting Figure 2.** RAD deficiency results in negative shift of activation curves of Ca_v_1.2 channels. The activation (A) and inactivation (B) curves in RAD^+/+^ and RAD^-/-^ mice.

**Supporting Figure 3.** Multiple alignment of Ca_v_1.2 α1 subunits in some species. The sequence of Ca_v_1.2 α1 subunits of rabbit, human, rat, mouse are aligned, in which the phosphorylated sites (Ser1700, Thr1704, Ser 1928) were marked in bold red.
