# Supplementary material for: Anchored PKA synchronizes adrenergic phosphoregulation of cardiac Cav1.2 channels
Source: J Biol Chem. 2024 Aug 10;300(9):107656. doi: 10.1016/j.jbc.2024.107656 (PMC11408856; doi:10.1016/j.jbc.2024.107656)
Supplement: Supplemental Figures S1–S3 [file mmc2.pdf]

Supporting Figure 1

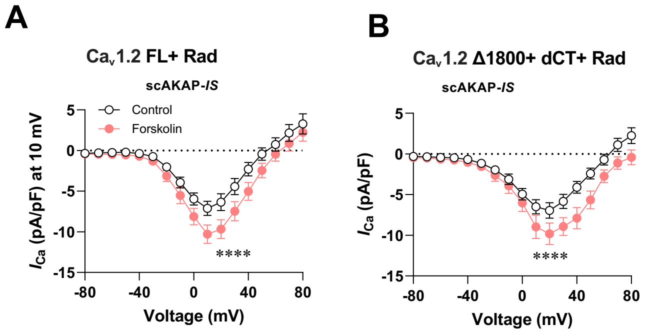

Sfig. 2

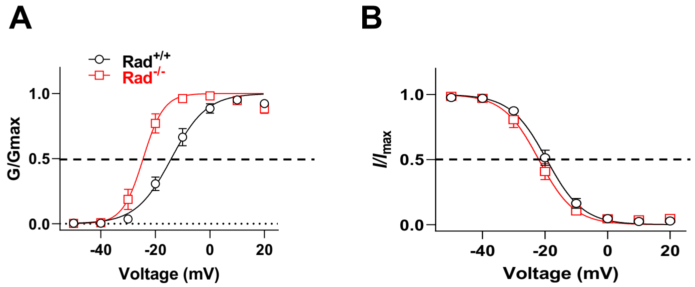
A
Rad+/+
Rad-/-
1.0
0.5
0.0
G/Gmax
-40
-20
0
20
Voltage (mV)
B
1.0
0.5
0.0
I/Imax
-40
-20
0
20
Voltage (mV)

Supporting Figure 3

	1700 1704	1928
Rabbit	1695 IRRAISGDLTAEEEL 1709	1923 LGRRASFHLEC 1933
Homo sapiens	1713 IRRAISGDLTAEEEL 1727	1976 LGRRASFHLEC 1986
Rat	1694 IRRAISGDLTAEEEL 1708	1922 LGRRASFHLEC 1932
Mouse	1665 IRRAISGDLTAEEEL 1679	1892 LGRRASFHLEC 1902
