# Supplementary material for: Anchored PKA synchronizes adrenergic phosphoregulation of cardiac Cav1.2 channels
Source: J Biol Chem. 2024 Aug 10;300(9):107656. doi: 10.1016/j.jbc.2024.107656 (PMC11408856; doi:10.1016/j.jbc.2024.107656)
Supplement: Supplemental Table S1 [file mmc3.pdf]

Table 1. Summary on different dose of isoproterenol for regulation of $Ca_v1.2$ channel

Concentration of isoproterenol (μM)	Mouse model citation
0.3	Rad knockout mice (12)
0.2	4S/A Rad knock-in mice (19)
0.2	35 α_1c mutation (18)
0.1	S1928A knock-in mice (30)
0.003	Knock-in mutant mice (22)
